# An Enhanced Sensing Application Based on a Flexible Projected Capacitive-Sensing Mattress

**DOI:** 10.3390/s140406922

**Published:** 2014-04-17

**Authors:** Wen-Ying Chang, Chi-Chun Chen, Chih-Cheng Chang, Chin-Lung Yang

**Affiliations:** 1 Department of Electrical Engineering, National Cheng-Kung University, Tainan 701, Taiwan; E-Mails: ee78350526@gmail.com (W.-Y.C.); chichun19771007@gmail.com (C.-C.C.); 2 Department of Pulmonary Medicine, Taipei Medical University Shuang-Ho Hospital, Taipei 235, Taiwan; E-Mail: 09005@s.tmu.edu.tw

**Keywords:** flexible projected capacitive-sensing technologies, charge time, guard ring, health care mattress

## Abstract

This paper presents a cost-effective sensor system for mattresses that can classify the sleeping posture of an individual and prevent pressure ulcers. This system applies projected capacitive sensing to the field of health care. The charge time (CT) method was used to sensitively and accurately measure the capacitance of the projected electrodes. The required characteristics of the projected capacitor were identified to develop large-area applications for sensory mattresses. The area of the electrodes, the use of shielding, and the increased length of the transmission line were calibrated to more accurately measure the capacitance of the electrodes in large-size applications. To offer the users comfort in the prone position, a flexible substrate was selected and covered with 16 × 20 electrodes. Compared with the static charge sensitive bed (SCSB), our proposed system-flexible projected capacitive-sensing mattress (FPCSM) comes with more electrodes to increase the resolution of posture identification. As for the body pressure system (BPS), the FPCSM has advantages such as lower cost, higher aging-resistance capability, and the ability to sense the capacitance of the covered regions without physical contact. The proposed guard ring design effectively absorbs the noise and interrupts leakage paths. The projected capacitive electrode is suitable for proximity-sensing applications and succeeds at quickly recognizing the sleeping pattern of the user.

## 1. Introduction

Diversified health products have been rapidly developed in recent decades. However, mattresses that influence the sleeping quality of people have not been extensively studied [1,2]. Medical mattresses can measure the patient's respiration, pressure distribution, decubitus posture [3], and sleeping activities [4–9]. These pressure-sensing measurements can also be used for other health care purposes such as the prevention of pressure ulcers [10,11] as well as monitoring of stumbling when exiting the bed and sleeping disorders [12,13]. Alternatively, several studies have used energy-transmitting devices to obtain data by observing static-charge variations [8,14].

Pressure can be measured from voltage variations to obtain piezo-resistive [15] or piezo-capacitive properties [16]. There are numerous differences between piezo-resistive-sensing methods and projected capacitive-sensing methods. The piezo-resistive-sensing method measures the resistance values that vary with the external contact pressure. The popular piezo-resistive-sensing device is a highly sensitive force-sensing resistor (FSR). Medical mattresses can also be used to measure respiratory and heart rate signals [17]. For piezo-resistive pressure sensors to correctly sense pressure, the sensors must be force receptors. If the force is only delivered but not received, the sensor cannot detect the correct pressure. Flexible substrate pressure sensors may cause invalid pressure-sensing results. This problem can be resolved by increasing the substrate strength of the pressure sensor so that it receives more force, and the pressure-sensing capability is enhanced. However, the design will cause discomfort during use. Over time, the sensing accuracy of the FSR will decline; this is one of the major limitations of FSRs.

**Figure 1.**
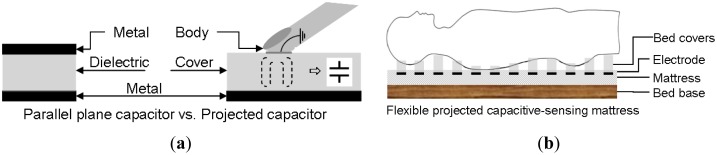
(**a**) Structure comparison of parallel plane capacitor and projected capacitor; (**b**) Flexible projected capacitive-sensing mattress.

The projected capacitive-sensing response result is determined by estimating the capacitance values. The most common application of projected capacitive-sensing technology is in consumer touch-sensing systems, where a single control variable is emphasized and the others are restricted to enhance the sensing accuracy. Projected capacitors have several advantages, including a high resistance to aging, simple components, and a low cost. The capacitance is mainly affected by three control variables. The common parallel-plane capacitor Equation (1) is used to explain the relationship among the three control variables *ε*, *A*, and *d*: For a projected capacitor, *d* is the thickness of the cover, structure comparison as shown in Figure 1a. When a parallel-pane structure transforms into a projected capacitive-sensing type, Equation (1) must be modified into Equation (2). For controllable applications, some variables must be fixed depending on the selected application. For example, if the area and the dielectric are constant, as presented in this paper, the capacitance can be related to the distance on proximity detector fields:

(1) C=ε×A/d

where *ε* is the dielectric constant of the material, *A* is the active area and *d* is the thickness between the metal planes. For a projected capacitor, *d* is the thickness of the cover, structure comparison as shown in Figure 1a. When a parallel-pane structure transforms into a projected capacitive-sensing type, Equation (1) must be modified into Equation (2). For controllable applications, some variables must be fixed depending on the selected application. For example, if the area and the dielectric are constant, as presented in this paper, the capacitance can be related to the distance on proximity detector fields:

(2) $$C=C_{\text{primary}}+\Delta C;\Delta C=C_{\text{primary}}\times (\frac{\partial C}{\partial \varepsilon }\Delta \varepsilon +\frac{\partial C}{\partial A}\Delta A+\frac{\partial C}{\partial d}\Delta d)$$

where *C _primary_* is the initial capacitor value before sensing.

In general, capacitive-sensing technologies respond more sensitively than piezo-resistive-based technologies. However, projected capacitive-sensing technology is also sensitive to interference in open environments, which influences the accuracy of the sensing results. Therefore, in this paper, several approaches are presented to overcome these interference issues and achieve the desired precision.

The present study discusses the development and properties of a projected capacitance-sensing device and the primary capacitance values that are derived from the electrode design. A sensory application method is further developed for large-area sensing to select an ergonomic, comfortable, and flexible substrate and to apply the projected capacitance-sensing technology in mattresses. This paper is structured as follows: Section 2 introduces the capacitance-sensing method, describes an experiment focused on the control variable of the primary capacitance values of the proposed flexible projected capacitive-sensing mattress (FPCSM), and presents the measurement results in details. Section 3 describes the development of the FPCSM. Section 4 presents the sensing results of the FPCSM and Section 5 provides a discussion about the FPCSM.

## 2. Methods and Capacitive Properties

**Figure 2.**
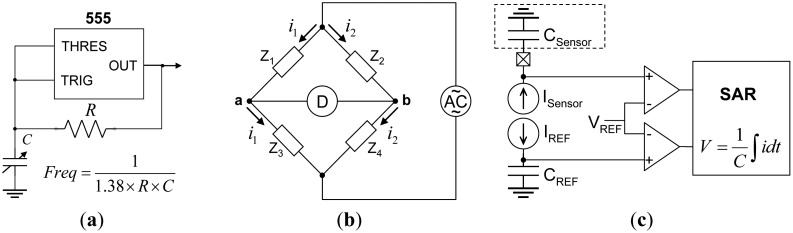
The capacitive-sensing methods: (**a**) the oscillation counting method; (**b**) the AC bridge method; and (**c**) the CT method.

Regarding capacitive-sensing technology, the two most commonly employed methods are the oscillation counter and the alternating-current (AC) bridge [18,19]. Figure 2a shows the first method, where several charge/discharge cycles are performed to complete the capacitance test by counting the number of oscillations. The function can be easily processed using a logic circuit. This method is economical but less time-efficient and less accurate than the second method. To obtain high accuracy, a longer processing time is required. By contrast, although the AC bridge method has an increased structure and operation complexity, it produces highly accurate results whose error rate is typically less than 1%. Figure 2b shows the AC bridge structure. Unlike these two methods, the charge time (CT) method [20], which is a rapid capacitance-testing technique, can be employed to complete capacitance tests within fewer charge/discharge cycles, which greatly reduces the operation time. The CT method requires only 38 μs to complete one capacitance measurement for each electrode. Thirty-two electrode capacitance measurements can be completed in less than 2 ms, which indicates a quick system response time. The resolution of the capacitance measurement can reach 1 femtofarad [20]. The operation structure of the CT method is shown in Figure 2c. The test target was charged with a constant current in a fixed, short period of time to increase the electrical potential. The accuracy of the timer counter should be considered for capacitance estimation. The equation for charging is a typical integration relationship as Equation (3):

(3) V(t)=(1/C)×∫idt

where *V(t)* is the voltage of capacitor, *C* is the value of capacitor and i is the charge current.

Subsequently, an analogue-to-digital converter (ADC) was employed to convert the capacitance measurements into digital values. Because a large number of the capacitance sensors were placed in the mattress, examining the capacitance of every electrode would be extremely time-consuming. Thus, slow-response technologies are not suitable for the proposed systems. Moreover, several concerns regarding the wired transmission of sensory signals must be taken into account, including the weak analog signal in the transmission that is vulnerable to interference, the quality degradation of the sensory signal by multiplex-channel switching, and the increased complexity of the system. These problems can be resolved by using a single-chip microcontroller with built-in CT testing functionalities [20]. This microcontroller enables rapid testing of the micro-capacitance and subsequent multi-capacitor sensing, substantially reduces the number of required external components, and lowers the implementation cost. The capacitance-testing functionality is built into the chip and can measure the testing capacitance up to 300 pF.

Because the CT method to measure the capacitance is limited by the upper bound of the chip specifications, the user of the CT method must pay attention to the sensed capacitance values to avoid going out of range. It is simple and effective to maintain a small initial electrode capacitance so that the detectable range of the sensing variation can be extended. The following section validates the projected capacitive characteristics and investigates the parameters that must be determined before the FPCSM system can be built. The measured results of the CT method and commercial LCR meters are compared. The primary capacitance value of the electrode provided a reference for design.

### 2.1. Correlation between the Primary Values and the Sensory Areas of the Electrodes and Validation of the CT Method

**Figure 3.**
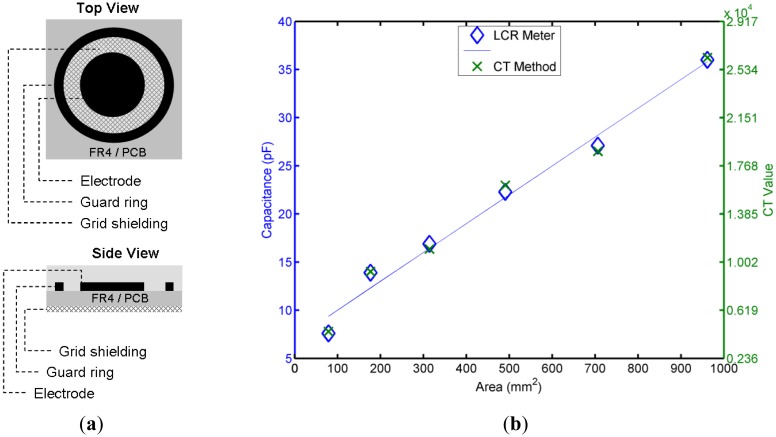
(**a**) Projected capacitive electrode; (**b**) Measuring and comparing the capacitance values of the electrodes with various diameters using the CT method and an LCR meter.

Like conventional capacitive devices, a projected capacitor suffers the drawbacks of leakage currents. Surface guarding or isolation is one solution that interrupts the leakage paths. Based on this concept, the projected capacitive electrode uses a guard-ring design. The capacitances of the electrodes with various diameters, which were measured using the proposed CT method, are compared with the values obtained using an ESCORT ELC-133A precision digital multi-meter. A Silicon Lab C8051F700, a microcontroller that can perform the CT method, was selected to measure the capacitance of the electrode samples. For the projected capacitive electrode test samples, an FR4 printed-circuit-board (PCB) substrate with a thickness of 1 mm was used, and seven projecting circular electrode samples of different diameters were designed and tested. Under identical conditions, a guard ring with a fixed separation distance and grid shielding on the backside was applied, as shown in Figure 3a. To calibrate the influence of the transmission line length, the numerical values obtained from the two capacitive-sensing methods were compared. The results after elimination of the initial primary values are presented in Figure 3b. The correlation between the two groups of values was greater than 99.7%, which indicates that the CT method can be used to determine the capacitance with highly accurate results. Because of the grid shielding, the electrode and the shielding grid form a parallel plate-type capacitor. Therefore, the electrode area and the capacitance value increase linearly.

### 2.2. Correlation between the Primary Values and the Sensory Areas of the Electrodes and Validation of the CT Method

**Figure 4.**
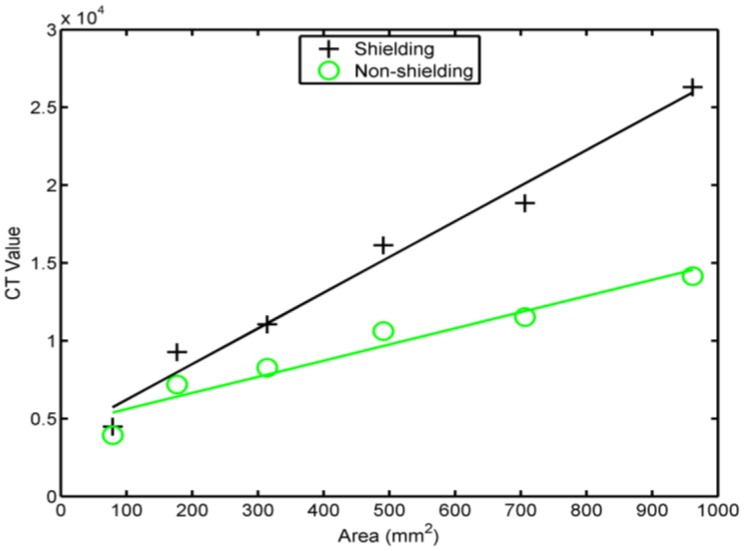
The variations of the projected capacitive-sensing substrates with and without ground shielding.

Prior to the capacitance testing, the primary values of the sample sensory electrodes should be considered and are denoted as the primary capacitance values, *C_primany_*; micro-variations in results of electrodes with large primary values may degrade the measured results and reduce the accuracy. The primary capacitance value increases proportionally to the electrode area. Therefore, the influence of the electrode area on the primary value should be calibrated during the subsequent design of capacitive-sensing tests. After removing the guard ring and the grid shielding, which absorbs and shields noise, the sensory electrode samples were tested, and the influence of the line transmission length was ignored. Figure 4 shows the obtained CT values which are the readout values of the ADC in CT method and are proportional to the capacitance. The constant scaling factor can be removed by a proper calibration procedure. When the electrode area increased, the influence of the shielding on the primary value also increased. Thus, the shielding must be carefully selected to limit the subsequent influence on the primary capacitance values of large-area electrodes. For the grid shielding experiments, the electrode had a similar structure to a parallel-plate capacitor. Without the grid shielding, the original electrode was only a single electrode without another parallel plate serving as a capacitor. Formulas of their capacitance are different and are expressed in Equations (1) and (4). In the formulas, the relationship between the capacitance and the electrode radius varies. Figure 4 shows the experimental results. Under identical variations in the electrode area, the capacitance variation ratio of the electrodes with shielding was different from that without shielding. In this paper, the grid shielding was removed because of the small primary capacitance values, which prevented signal detection in the following stages due to saturation. The guard ring technology minimizes primary capacitance value increases and interrupts leakage paths:

(4) $$C=3.54\times 10^{-12}\times \varepsilon_{r}\times D$$

where *D* is the electrode diameter (in meters).

### 2.3. Transmission Line Length Influences the Primary Capacitance Values

**Figure 5.**
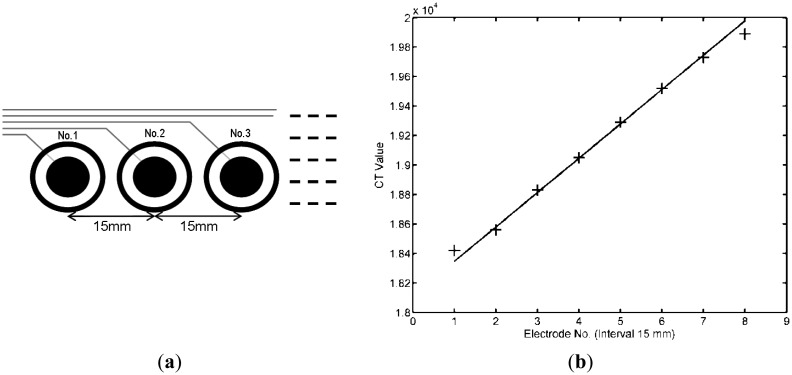
(**a**) Electrode arrangement in the test sample; (**b**) The CT measured value of identical-size electrodes with different transmission lengths with and without shielding.

The transmission line length affects the primary capacitance value and should be removed initially. An experiment focused on the transmission line length was conducted at the initial stage. In this experiment, FR4 PCB was selected as the board material, and the electrodes were arranged at fixed separations (at 15 mm from the center of the neighboring electrodes), as shown in Figure 5a. The same design was used to maintain the electrode conditions except for the varying transmission line lengths. Figure 5b shows the primary capacitance values of the sensory electrode samples that were determined using the CT method. The results show that the transmission line length indeed influences the primary capacitance value: an increase in the transmission line length causes a linear increase in the primary value, except for the first and eighth electrodes. These results occurred because the first and eighth electrodes were near the circuit board edge, so their values did not match the linear arithmetic progression. This result indicates that a shorter transmission line results in lower capacitance values. In the case of small electrode capacitance differences, the length of the transmission line should be carefully designed in the circuit layout because differences in length affect the capacitance values. Therefore, shorter connecting transmission lines were used in our design.

### 2.4. The Variation of Capacitance with Pressure

**Figure 6.**
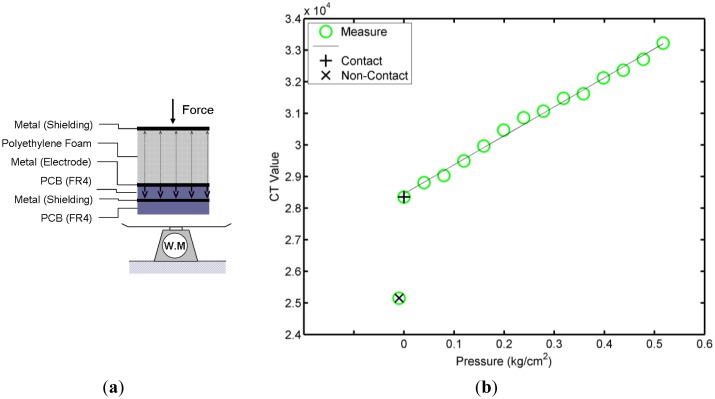
(**a**) The diagram of the experiment; (**b**) The CT measured capacitance with pressure.

The experiments revealed the relationships between the projected capacitance value and the varying pressure. The experimental schematic setup is shown in Figure 6a. To remove the effects of the metal plate of the weight meter, a 1-mm-thick FR4 material was placed on the metal plate. Moreover, a metal plate that connected to the ground was placed on top of the polyethylene foam. The top of the electrode, a 2-mm-thick polyethylene foam layer, functioned as a coupled insulating medium and constituted a parallel-plate capacitor with the grounded metal plate. The medium of this parallel-plate capacitor was compressible. When the medium was compressed by an external pressure, the distance and the dielectric changed the capacitance value of the electrode sensor. Figure 6b shows the experimental results. The electrode capacitance varied linearly with the pressure in the experimental test range. As the pressure increased, the capacitance also increased. Figure 6b shows the non-contact value, which indicates that there was no metal on the polyethylene foam, whereas the contact indicates that there was metal on the polyethylene foam. The CT value changed significantly when there was no pressure; this was the primary difference between the pressure sensing and the projected capacitive sensing. The projected capacitive sensing could sense whether there was a conductor without pressure.

Based on the experiments described above, three design parameters that influenced the projected primary capacitance values were identified: the electrode area, the ground shielding, and the transmission line length. These parameters exhibited various degrees of influence on the primary capacitance values. To further develop the FPCSM, the grid shielding should not be used, but guard rings are preferred. Moreover, the length difference of the transmission line should be minimized.

## 3. Description and Development of the Proposed System

**Figure 7.**
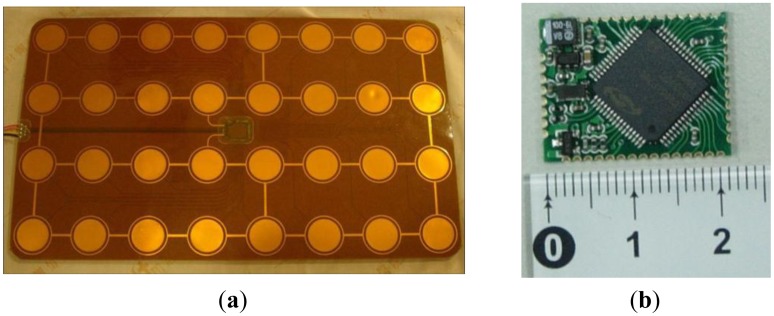
(**a**) Sensory electrodes on a 40 × 25 cm^2^ flexible substrate; (**b**) CT sensing sub-module.

The proposed FPCSM consists of three main modules: sensory electrodes, CT sensing sub-modules, and a coordinator, as shown in Figure 7a. The coordinator uses 10 CT sensing sub-modules, each of which is designed with 32 capacitive sensory electrodes. These three components constitute a 0.8 × 1.2 m^2^ FPCSM unit. The FPCSM was covered with cloth. The functional properties of each component are discussed in the following sections.

### 3.1. Sensory Electrodes

Several potential problems that affect the measurement accuracy were considered with respect to the sensory electrodes: the interference caused by external electricity noise, mutual interference, and the physical properties of the structure. The selected material and design should aim to reduce these electric problems. First, to reduce the interference caused by leakage current and external noise, a guard ring was used to absorb the external interference and reduce the interference coupled to the electrodes. Although this design increased the primary capacitance values of the electrodes and utilized part of the dynamic range for available sensing, subsequent sensory trends were not influenced. In addition, to ensure that the capacitance variation was mostly induced by the electrodes, the influence of the transmission line needed to be reduced. Therefore, a thin transmission line design was selected. The width of the transmission line was 0.2 mm. It is difficult to design all transmission lines such that the length from the electrodes to the capacitive-sensing microcontroller remains identical. To minimize length differences among transmission lines, the microcontroller can be placed at the center of sensory electrodes for design symmetry.

To prevent capacitive coupling interference, which is caused by a line arrangement, the layout of the transmission line gap was over seven times wider than the line width. For improved comfort of the user (while lying on the electrode), a flexible polyimide PCB was selected. The flexible PI PCB was 25 μm thick with a 12.5 μm PI insulating protective layer on both the top end and the bottom end; this layer covered the transmission lines to protect them. The resilience of the PI PCB, designed to prevent transmission line breaks, was shown to appropriate for experiments and regular use.

The data obtained from the previous experiments were used as a reference to re-design the electrode size. Due to the limitation of the fabrication machine size (45 × 30 cm^2^), the PCB that contained 32 electrodes was enlarged as much as possible (40 × 25 cm^2^, as shown in Figure 7a).

### 3.2. CT Sensing Sub-Modules

**Figure 8.**
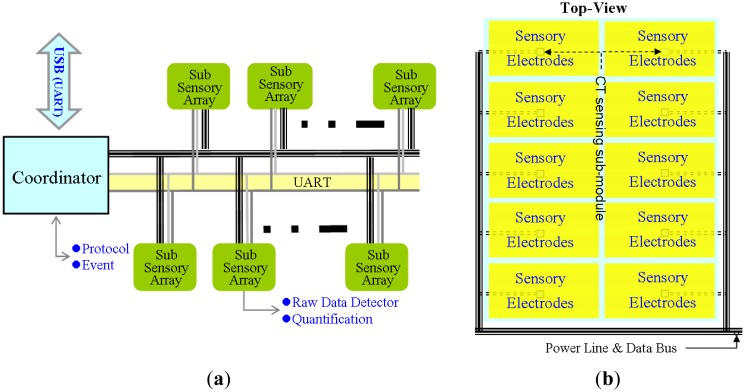
(**a**) Connection of the FPCSM; (**b**) Top view of the FPCSM.

The main component of the CT sensing sub-module had a capacitive-sensing microcontroller and a low-drop-output (LDO) regulator. The size of each sub-module was 22 × 17 mm^2^, and their main functions are listed as follows: (1) constant monitoring of the capacitance and periodic updating of the sensory results; (2) intercepting the bus packets using a 9-bit universal asynchronous receiver/transmitter (UART) protocol with an identification (ID) functionality to intercept the packets that match the sub-module ID overhead, thereby effectively reducing the operational load of the microcontroller; (3) executing the communication packet protocol by sending communication packets to the coordinator to introduce the checksum mechanism and limiting the transmission time to avoid transmission packet errors; (4) computing the measured values as binary results to reduce the follow-up data processing load; and (5) maintaining the basic operations of the sub-module and avoiding unnecessary interruptions. In addition to a watchdog timer, a communication-timeout-and-reset mechanism was employed to ensure normal operation of the sub-module, as shown in Figure 8b.

### 3.3. Coordinator

The main purpose of the coordinator is to serve as the intermediary between the sensors and the control PC. This study utilized an adjustable rate and different data link protocols in the system to transmit the external and internal data. The coordinator must maintain system coordination of internal/external data packet transmission and conversion. The external and internal communication protocols are described below:

(a) An 8-bit UART protocol was used for external (upload) communication, which allowed the coordinator to link with the PC at a rate of 230,400 baud. Because of the convenience of bit control in a PC environment, the common 8-bit protocol was selected.
(b) A 9-bit UART protocol with ID functionality was used for internal (download) communications, thus allowing the coordinator to transmit bidirectional information within its allocated CT sensing sub-modules at a high-speed rate of 460,800 baud. Using this additional 9-bit system, the heavy communication load of the single-chip microcontroller can be effectively reduced to collect multiple capacitance-sensing results. Therefore, the high-speed transmission can effectively reduce the data transmission time in the communication bus even when the amount of data transmitted within a fixed period increases. A check code was incorporated into the communication packets to ensure the completeness of every packet and to prevent transmission failure. Although the use of a check code improves the data communication quality, the amount of effective data transferred is reduced because of the redundancy overhead. To avoid this drawback, the check code proportion in each packet was minimized.

## 4. Experimental Results

Regarding the experiments, the proposed system was built and placed on a spring bed, and the coordinator implemented bidirectional data transmission to the PC at a rate of 230,400 bauds. The experiments were conducted in two stages: determination of the FPCSM primary value and the actual user tests. The first stage measured the initial capacitance before using the capacitive-sensing system, and the second stage examined the capacitance distribution variations of the participants placed in a decubitus position on top of the system.

**Figure 9.**
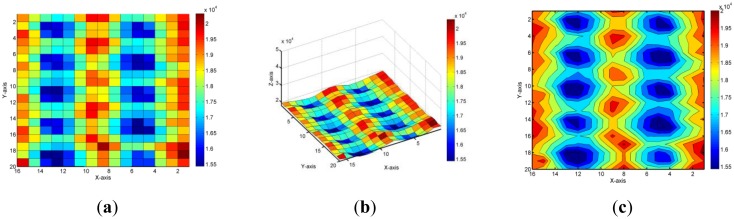
Primary capacitance values of the projected capacitance-sensing mattress. (**a**) Top view; (**b**) Side view; (**c**) Contour map.

Figure 9 shows the initial primary value of an FPCSM with 10 CT sensing sub-modules for the first stage, prior to calibration and sensing, when there is nothing placed on the FPCSM, as shown in Figure 9a. The minimum capacitance CT values were over 1.5 × 10^4^. The difference between the maximum and minimum capacitance CT values reached 5,000. The CT Value 5,000 is set as the representative of capacitance value 6.5 pF in this system. The major cause of these differences was the influence of the transmission line length on the CT values, as explained in Section 2. The minimum length of the transmission line was less than 2 cm. The maximum length of the transmission line from the electrode to the capacitive-sensing microcontroller was more than 22 cm. The data distribution is shown in a 3D diagram in Figure 9b. Ten concaves were observed, representing the relative minimum capacitance of each CT sensing sub-module. In the concave regions, the transmission lines that were connected to the sensory electrodes and the capacitive-sensing microcontroller were comparatively shorter than those in the other regions. In Figure 9c, the contour map displays regular change according to the transmission line length for each module. The layout of the transmission line lengths was carefully implemented with a symmetrical design.

**Figure 10.**
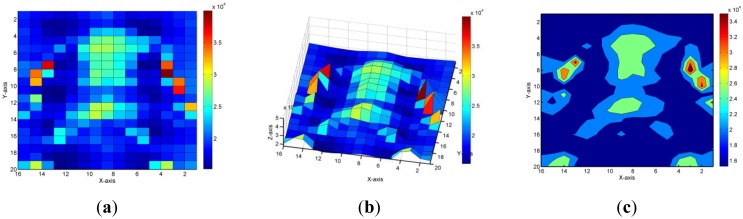
The sensory map of a participant lying on the FPCSM in a supine position. (**a**) Top view; (**b**) Side view; (**c**) Contour map.

Figure 10 displays the data that were recorded when one participant was lying supine on the FPCSM. The subject's clothing and the packaging of the FPCSM were located between the body and the sensing electrodes. They were both made of a cloth material that was identical to the cover shown in Figure 1a. Because most people place their head on a pillow when they sleep, the head portion was not detected [3,11]. The thickness of the FPCSM packaging was less than 1 mm, and the thickness of included the subject's clothing and the FPCSM packaging was less than 2 mm without pressure. The subject's limbs were not covered with any cloth material. In Figure 10, the body foci of the supine subject (*i.e.*, the torso, the arms, and the legs) can be clearly recognized. The contour graph clearly presents the sleeping posture based on the measured results. The sensory capacitance distribution of the body on the mattress varies according to the different positions and regions. Because the body surface is curved, the lumbar area and the joints do not touch the mattress. The pressure principle cannot be used to sense those regions of the body that do not come into contact with the mattress. The FPCSM applies the method of projected capacitive sensing, which detects pressure in both situations. Therefore, even for a rigid mattress, the lumbar and the joint areas can be detected according to changes in capacitance.

**Figure 11.**
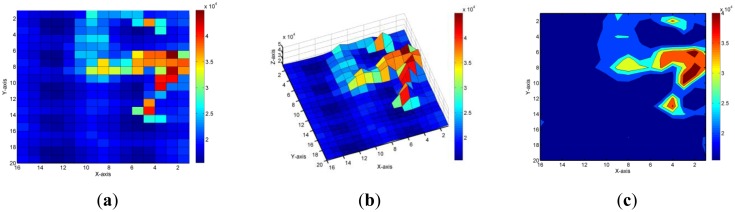
A sensory map of a participant lying on the FPCSM in the right lateral position. (**a**) Top view; (**b**) Side view; (**c**) Contour map.

The prone position can be observed clearly using visual identification. Figure 11 shows the sensory map of a participant lying on the FPCSM in the right lateral position. Because the FPCSM had 320 sensing electrodes in the experiment, the lying position could be visually identified easily. As shown in Figures 10 and 11, the capacitance sensing values of the limbs appeared larger than those of the torso. The pressure of the torso was greater than that of the limbs when the pressure-sensing mattress was used. The FPCSM appears to outperform the pressure-sensing method. Because of different sensing principles, the FPCSM could sense the capacitance change of the projected capacitors, which was determined by the three related control variables. The thickness of the cover was reduced because of external pressure. However, the thickness of the cover under the limbs was less than that of the cover under the torso. Some of the sensing capacitor values of the limbs were larger than those of the torso. Therefore, the FPCSM provides an alternative sensing technique using the projected-capacitor principle.

## 5. Discussion

Regarding the experimental results, the properties and the configuration structure of the capacitors depended on the electrode design. Before posture sensing, the sensory electrode was a single electrode with a simple configuration consisting of a single thin plate. For the capacitors of this single-plate electrode, the reference ground did not exit and was regarded as infinite. In this configuration, the primary capacitance value of the electrode increases linearly with the electrode diameter, as observed in Equation (4). In addition, the capacitance value of a single electrode increases with an increase in transmission line length.

When a body is placed on the FPCSM, the body replaces the distant, infinite ground because the body is close to the electrode. The configuration of the sensory electrode is no longer a single electrode (right part of Figure 1a) but rather two parallel conductive plates (left part of Figure 1a) with permittivity ε of the covering cloth). The capacitance structure becomes a parallel-plane capacitor. In this case, the control variables of the FPCSM are identical to those of a parallel-plate capacitor.

Therefore, the control variables of the parallel-plate capacitor for sensing with the FPCSM should be examined further to improve the FPCSM system. Increasing the size of the sensing area and reducing the area of the transmission line were shown to be simple and effective methods to control the area variables. Two methods were used to reduce the area of the transmission line: thinning the trace width and shortening the length of the transmission line between the electrode and the sensory chip.

The cover thickness is a denominator variable in the parallel-plate capacitor equation. The contribution of the capacitance variation to the cover thickness depends on the initial value of the cover thickness. To remove that variable, an incompressible material was chosen for the cover, which increased the sensing sensitivity. By conditionally constraining the cover thickness, one can improve the sensing contribution and maintain a high sensitivity. The cover of the FPCSM is composed of the FPCSM packaging and the participant's clothing. The packaging of the FPCSM is thin and cannot be compressed, but the clothing of the participant can be compressed and affect the measurement results. Comparison of various sensing techniques was displayed in Table 1.

FPCSM sensing, which occurs in an open environment, relies on the characteristics of capacitance variation. Either a single or a dual electrode configuration can be used for proximity detection. Unlike an FSR-based pressure-sensing mattress, the FPCSM can apply the capacitance-sensing technology to non-pressure objects. No existing method allows pressure-sensing mattresses to detect such non-pressure objects. The guard ring design for FPCSM electrodes can effectively absorb noise, align the electric field that is perpendicular to the electrode surface, and interrupt the leakage paths. The guard ring can also make the electrode more suitable for proximity-sensing applications.

## 6. Conclusions

This research introduced the FPCSM system, which consists of a microcontroller with built-in CT capacitance measurement technologies. A single electrode capacitance measurement can be performed in only 38 μs, and the capacitance measurement of the entire 32-electrode system can be performed in less than 2 ms with a high resolution of 1 femtofarad. Compared with using a precision digital multi-meter, a correlation coefficient of up to 99.7% can be achieved using the FPCSM system. Using additional shielding will increase the primary capacitance value of the electrode. The CT sensing sub-module has a limited sensing range. The low primary capacitance electrodes retain a more dynamic range of sensing variation. It was confirmed that the length from the sensing electrodes to the microcontroller input will influence the primary capacitance value. When the transmission line length increases, the primary capacitance value of the electrode also increases. To reduce variations in the primary capacitance value of each electrode, a long wire length is preferred, although it will reduce the dynamic range available for sensing. An FPCSM sensing system was implemented using a PI substrate, which was flexible. The FPCSM was composed of 320 sensing electrodes, and the overall size was 0.8 × 1.2 m^2^. The proposed system records data for a body lying on the mattress using projected capacitance sensing. It can visually and clearly identify the sleeping posture of the user without requiring a complicated algorithm. The FPCSM has the advantages of a low cost, a high resistance to aging and non-contact capacitance detection. The application of projected capacitive sensing to the field of health care is novel. The proposed FPCSM system is suitable for sleep studies and can be applied to sleep-related health products.

## Figures and Tables

**Table 1.** Performance comparison with published works.

	**SCSB** [2]	**BPS** [16]	**FPCSM**
Theory	Static Charge	Pressure Transducer	Projected Capacitor
Variable	Volume	Pressure	Static electric fields
Feature	Vibration detected	Force sensing	Proximity sensing
Pads Cost	Low	High	Low
Aging Resistance	High	Medium	High
Applications	Body movements/Respiration/BCG	Body movements/Respiration/Posture	Body movements/Posture

SCSB: Static Charge Sensitive Bed; BPS: Body Pressure system.			
